# A Silicone-Based Film-Forming Gel Wound Dressing for Radiation Dermatitis in Head and Neck Cancer Patients: A Retrospective Cohort Analysis Using Clinical Informatics

**DOI:** 10.7759/cureus.103220

**Published:** 2026-02-08

**Authors:** Eulanca Y Liu, Erika Jank, P. Travis Courtney, Jesus Juarez, Ting Martin Ma, William Delery, Lydia Chau, Vishruth Reddy, Myung Shin Sim, Robert K Chin, Ricky R Savjani

**Affiliations:** 1 Radiation Oncology, University of California Los Angeles, Los Angeles, USA; 2 Radiation Oncology, Physics and Biology in Medicine, University of California Los Angeles, Los Angeles, USA; 3 Radiation Oncology, University of Washington, Seattle, USA; 4 Radiation Oncology, Neurological Surgery, University of California Los Angeles, Los Angeles, USA; 5 General Internal Medicine and Health Services Research, University of California Los Angeles, Los Angeles, USA

**Keywords:** desquamation, film-forming gel, head and neck cancer (h&n cancer), radiation dermatitis, retrospective clinical trial

## Abstract

Purpose

Patients undergoing radiotherapy for head and neck cancers often experience radiation dermatitis, which is typically managed by applying topical moisturizers. A silicone-based polymer film with antibacterial properties, StrataXRT, has been developed to mitigate radiation-induced side effects. We sought to determine the efficacy of StrataXRT compared with standard moisturizers in reducing the incidence of severe radiation dermatitis in patients with head and neck malignancies.

Materials and methods

We conducted a retrospective analysis of patients treated with StrataXRT compared with historical controls using conventional moisturizers (largely Aquaphor®) via a clinical informatics approach. Propensity score matching was used to control for dosimetric properties: mean skin dose, maximum skin dose, and the surface area receiving 40 Gy or higher. The endpoint was grade 2+ radiation dermatitis, measured using Common Terminology Criteria for Adverse Events or CTCAE, in 171 patients treated with StrataXRT, compared with 171 matched patients treated with a standard moisturizer. Multivariable Cox regression was performed to estimate the association between StrataXRT and standard moisturizer while adjusting for confounding clinical variables.

Results

In the multivariable Cox regression, the hazard ratio was 0.61 (95% CI (0.37, 1.0), p=0.049) with a relative reduction in the risk of grade 2+ radiation dermatitis of 36.5% with the use of StrataXRT compared with standard moisturizers. The absolute risk reduction with StrataXRT was 14.5%, which translates to a number needed to treat of seven patients to prevent one occurrence of grade 2+ radiation dermatitis.

Conclusions

Patients benefited from StrataXRT, with a significant reduction in the incidence of grade 2+ radiation dermatitis throughout the course of treatment. This study supports the adoption of a novel topical agent providing barrier protection in the form of a film-forming gel to reduce acute radiation dermatitis in head and neck cancer patients.

## Introduction

One of the most common side effects of radiotherapy to the head and neck is radiation dermatitis, which affects around 85% of patients despite advances in radiation treatment techniques and clinical care [1,2]. Recently, a novel treatment option has been developed to address this issue. The silicone-based film-forming gel dressing StrataXRT adheres well to the skin in all body areas, does not have a bolus effect, and is waterproof. Prior prospective studies in breast cancer patients undergoing radiotherapy have shown that StrataXRT can reduce objectively measured physiological skin responses such as erythema, moist desquamation, and edema [3,4]. In the context of head and neck cancers, a single-blind randomized controlled study revealed a reduction in both grades 2 and 3 radiation dermatitis with StrataXRT compared with sorbolene, a glycerin-based moisturizer [5]. Two pooled meta-analyses have also indicated a potential benefit of StrataXRT in reducing grade 2+ dermatitis [6,7]. 

At our institution, we adopted StrataXRT starting in late 2020 for most of our head and neck cancer patients undergoing radiation. In this study, we sought to compare grade 2+ radiation dermatitis in patients receiving StrataXRT with our approach before 2020 of using standard moisturizers, largely Aquaphor®. To facilitate this retrospective analysis, we used a sandboxed copy (i.e., extracted data in an isolated environment designed for data science) of the entire medical record from which we extracted the clinical data for head and neck cancer patients treated with radiotherapy. This enabled us to conduct a real-world clinical informatics study in which relevant treatments, adverse effects, and clinical data were programmatically obtained from digital records.

Radiation dermatitis remains a complex toxicity among patients with head and neck cancers, often limiting the timely and successful completion of radiation. Although a few prospective studies indicate that StrataXRT may reduce the severity of radiation dermatitis, robust evidence from real-world clinical adoption is lacking, and thus, widespread utilization is limited. We hypothesized that upon adoption in our clinic, StrataXRT, compared with standard moisturizers, would show a significant reduction in the incidence of grade 2+ radiation dermatitis or a significantly delayed onset, even after adjusting for treatment-related confounders.

## Materials and methods

Study design

Institutional review board approval was obtained for this retrospective study with a waiver for informed patient consent. The retrospective study was registered as NCT05810194 on ClinicalTrials.gov.

Patient data extraction and clinical informatics

We worked with our institutional office of health informatics and analytics team to obtain a sandboxed copy of electronic medical record (EMR) patient data for patients treated with head and neck radiotherapy from 3/1/2013 to 4/1/2023. Data were housed in a secure Health Insurance Portability and Accountability Act (HIPAA)-compliant environment in the form of comma-separated variable files for each type of record (e.g., patient notes, appointments, encounters, medications, etc.). Patients were assigned to treatment groups based on the topical skin regimen received during the treatment course: control moisturizers (primarily Aquaphor®) vs. StrataXRT. Each patient was required to have at least five weekly on-treatment visit (OTV) notes documented to ensure that Common Terminology Criteria for Adverse Events (CTCAE) toxicity data were captured. Before 2017, CTCAE v4.0 [8] was used, and after 2017, v5.0 [9] was used. For radiation dermatitis, the definitions remained identical for both CTCAE v4.0 and v5.0 and are shown in Table 1. Additionally, our institutional template note used the same CTCAE scoring from 3/1/2013 to 4/1/2023. 

**Table 1:** CTCAE scoring for radiation dermatitis (identical for v4.0 and v5.0) CTCAE, Common Terminology Criteria for Adverse Events.

Grade	Severity
Grade 1	Faint erythema or dry desquamation
Grade 2	Moderate to brisk erythema; patchy moist desquamation, mostly confined to skin folds and creases; moderate edema
Grade 3	Moist desquamation in areas other than skin folds and creases; bleeding induced by minor trauma or abrasion
Grade 4	Life-threatening consequences; skin necrosis or ulceration of full-thickness dermis; spontaneous bleeding from the involved site; skin graft indicated
Grade 5	Death

The tabulated data were accessed programmatically using custom in-house scripts in Python (version 3.10.11; Python Software Foundation, Fredericksburg, VA, US). Notably, weekly OTV notes at our institution are templated. These notes contained structured text in which CTCAE toxicity scores and treatment regimens (including the particular skin regimen) were documented systematically for all patients through checkboxes. These data were extracted using string pattern matching. Further, treatment start and end dates were pulled from patient encounters. Final treatment summaries were collated for all patients to gather relevant oncological data such as surgical status, use of concurrent chemotherapy, and p16+ status. Lastly, demographic data (age, gender, ethnicity, smoking history, body mass index (BMI), and diabetes history) were systematically extracted for each patient. Manual chart review was performed for quality control and to fill in elements missing from the data extraction.

Propensity score matching on skin dosimetry

A potential confounder in comparing radiation dermatitis between patients treated with StrataXRT and those treated with standard moisturizers was the radiation dose the skin received. We first automated extraction of the mean skin dose, maximum skin dose, and the surface area receiving 40 Gy or higher (Skin_S40). We created a workflow in MIM Maestro (MIM 7.1.4; MIM Software, Cleveland, Ohio) to extract these dosimetric values for each patient’s planned radiation dose. The workflow loaded the clinical contour for the skin (3-mm thickness) and computed dosimetric values, including the skin surface area getting 40 Gy or higher, the maximum skin dose, and the mean skin dose. We created a Bash shell script to run the workflow and save dosimetric information for each patient.

To mitigate these confounders, we applied one to one propensity score matching to match each patient treated with StrataXRT to a matching patient treated with the control moisturizer, using the following dosimetric properties: mean skin dose, maximum skin dose, and the surface area receiving 40 Gy or higher [10]. We used the nearest method for greedy nearest-neighbor matching. The generalized additive model (GAM) was used for the distance function with calipers of 1 standard deviation for each of the three dosimetric properties. GAM allows nonparametric and nonlinear modeling to better balance StrataXRT and control patients on these dosimetric values [11]. Notably, the dosimetric variables used in propensity score matching were not subsequently used as covariates in regression analyses.

Statistical analyses

The primary endpoint was the development of CTCAE grade 2 or higher radiation dermatitis. All patients were aligned in time to the start of radiation. Multivariable Cox regression was performed using covariates, including the use of StrataXRT, age, gender, receipt of concurrent systemic therapy (none, chemotherapy, or cetuximab), surgical resection before radiation, race, smoking history, BMI, and diabetes history. The relative risk reduction was computed using Poisson regression with robust error variance [12]. The absolute risk reduction and corresponding number needed to treat were estimated over time within the survival analysis using the Austin method [13,14]. Lastly, two-sample, two-sided t-tests were used to compare demographics for continuous variables and χ2 tests of independence for categorical variables. All statistical analyses were conducted in R version 4.3.0 (R Foundation for Statistical Computing, Vienna, Austria, https://www.R-project.org/).

## Results

**Figure 1:**
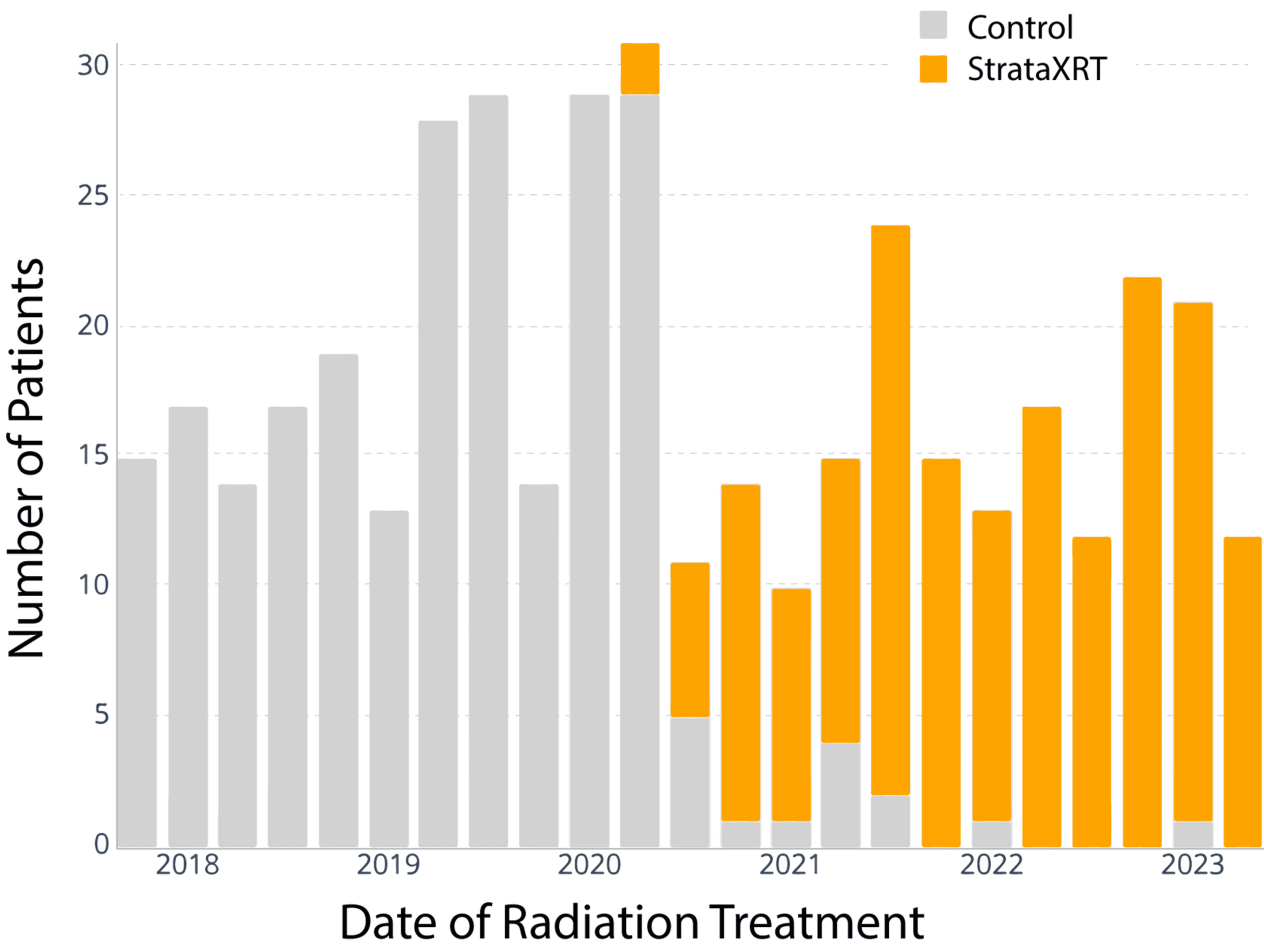
Number of patients treated with StrataXRT vs. control moisturizer by year StrataXRT was adopted in late 2020 for head and neck cancer patients undergoing radiation therapy at our institution.

Data from 171 patients treated with StrataXRT and 236 control patients were extracted from the EMR. Figure 1 shows our topical skin treatment usage patterns over time, highlighting the institution's adoption of StrataXRT in 2020.

Propensity score matching skin dosimetric properties allowed for the nearest matching of 171 of the 236 control patients with 171 StrataXRT patients. 132 (77.2%) of the matched control patients used Aquaphor®, as was our prior standard of care; the remaining 39 (22.8%) did not have the specific skin care agent documented, as their treatment occurred before the adoption of StrataXRT in the clinic.

**Figure 2:**
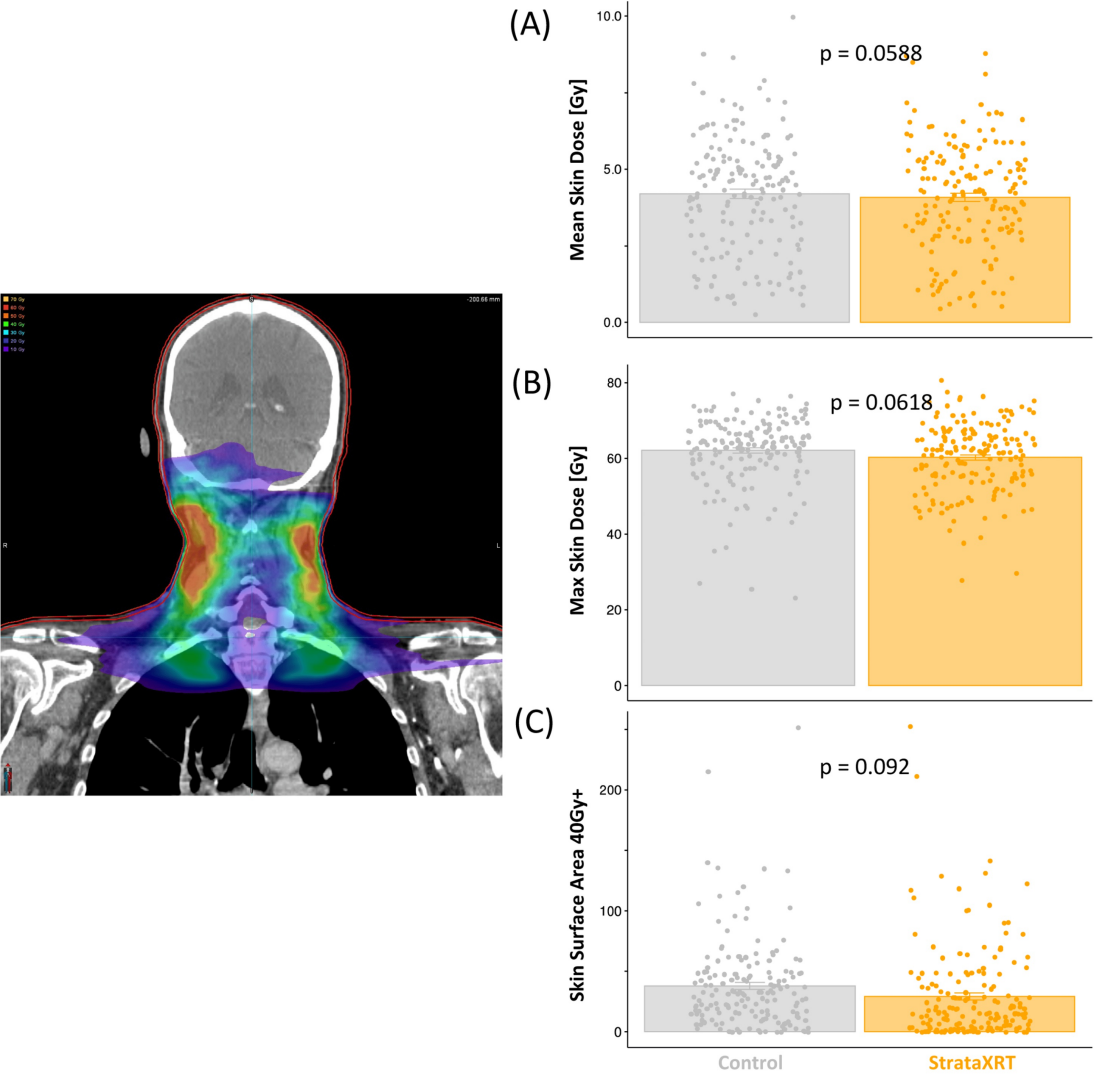
Skin dose distributions across control vs. StrataXRT groups (A) Mean skin dose, (B) maximum skin dose, and (C) surface area receiving 40 Gy or higher (Skin_S40) distributions across control and StrataXRT groups after propensity score matching show no statistical differences. The left image shows the dose distribution with a 3-mm skin contour in red.

Table 2 shows the demographic breakdown of patients in both groups, with significant testing between groups. Race, age, and disease site were significantly different between the two groups. Age and race were included as covariates in the multivariable Cox regression. The disease site was not directly included, as the skin dose was accounted for in propensity score matching. Figure 2 shows the distribution of dosimetric properties (mean skin dose, maximum dose, and Skin_S40) between the two groups after propensity score matching was applied. Notably, no dosimetric values were significantly different in the selected cohorts.

**Table 2:** Patient demographics *The p-value indicates whether there is a statistically significant difference between the two groups (control vs. StrataXRT). BMI, body mass index; SD, standard deviation.

Variable	Control (N=171)	Strata (N=171)	p-value
GENDER			1.000
Male	122 (71.3%)	121 (70.8%)	
Female	49 (28.7%)	50 (29.2%)	
AGE			0.003*
Mean (SD)	67.4 (13.4)	63.2 (12.4)	
Median (minimum, maximum)	68.0 (22.0, 98.0)	64.0 (24.0, 88.0)	
SURGERY			0.071
No	70 (40.9%)	53 (31.0%)	
Yes	101 (59.1%)	118 (69.0%)	
SYSTEMIC THERAPY			0.053
No	92 (53.8%)	91 (53.2%)	
Chemotherapy	64 (37.4%)	75 (43.9%)	
Cetuximab	15 (8.8%)	5 (2.9%)	
p16+			0.306
No	117 (68.4%)	107 (62.6%)	
Yes	54 (31.6%)	64 (37.4%)	
RACE			0.008*
American Indian or Alaska Native	0 (0%)	1 (0.6%)	
Asian	16 (9.4%)	21 (12.3%)	
Black or African American	5 (2.9%)	4 (2.3%)	
Do not identify with race	3 (1.8%)	7 (4.1%)	
Middle Eastern or North African	0 (0%)	7 (4.1%)	
Multiple races	6 (3.5%)	8 (4.7%)	
Native Hawaiian or other pacific islander	2 (1.2%)	0 (0%)	
Other	27 (15.8%)	43 (25.1%)	
Patient refused	10 (5.8%)	13 (7.6%)	
Unknown	5 (2.9%)	3 (1.8%)	
White or Caucasian	97 (56.7%)	64 (37.4%)	
SITE			0.016*
Oropharynx	61 (35.7%)	69 (40.4%)	
Oral cavity	29 (17.0%)	13 (7.6%)	
Skin	20 (11.7%)	36 (21.1%)	
Larynx	19 (11.1%)	14 (8.2%)	
Salivary gland tumors	13 (7.6%)	8 (4.7%)	
Thyroid	11 (6.4%)	7 (4.1%)	
Nasal cavity	7 (4.1%)	10 (5.8%)	
Nasopharynx	3 (1.8%)	5 (2.9%)	
Cancer of unknown primary	3 (1.8%)	0 (0%)	
Lymphoma	3 (1.8%)	8 (4.7%)	
Chordoma	2 (1.2%)	0 (0%)	
Pharynx	0 (0%)	1 (0.6%)	
SMOKING			0.923
Every day	1 (0.6%)	2 (1.2%)	
Former	62 (36.3%)	65 (38.0%)	
Never	107 (62.6%)	103 (60.2%)	
Some days	1 (0.6%)	1 (0.6%)	
BMI			0.566
Mean (SD)	26.9 (5.26)	26.5 (5.83)	
Median (minimum, maximum)	26.4 (16.7, 48.1)	25.4 (12.8, 62.1)	
DIABETES			0.863
No	153 (89.5%)	151 (88.3%)	
Yes	18 (10.5%)	20 (11.7%)	

**Figure 3:**
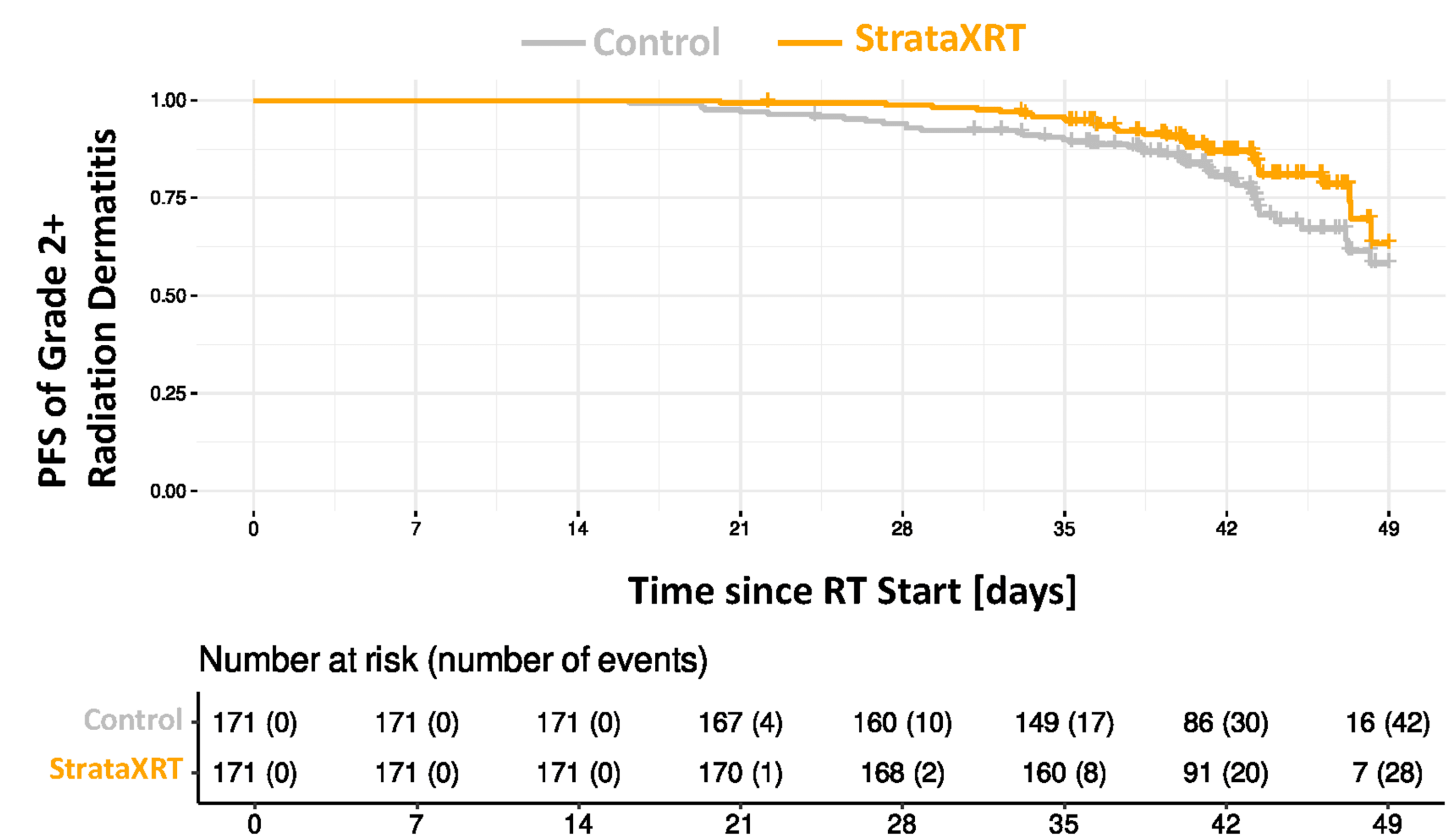
Kaplan-Meier progression-free survival (PFS) of grade 2+ radiation dermatitis in both control and StrataXRT groups

**Figure 4:**
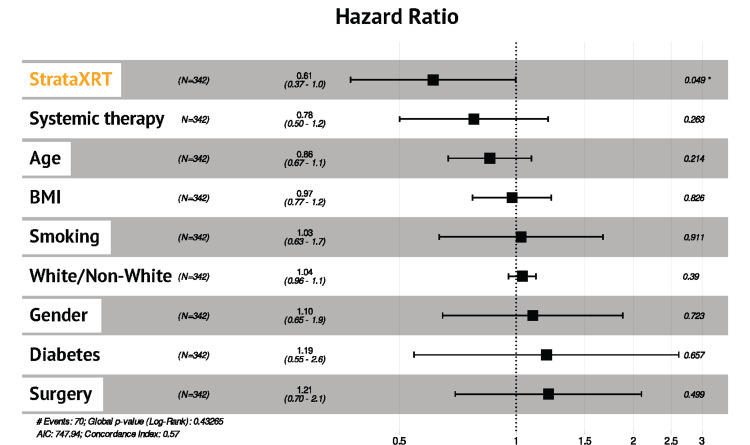
Hazard ratios and 95% confidence intervals for multivariable Cox regression analyses StrataXRT was the only variable significant in reducing the incidence of grade 2+ radiation dermatitis. BMI, body mass index.

Figure 3 shows the Kaplan-Meier survival curves for the development of grade 2+ radiation dermatitis for both the StrataXRT and control groups. In the multivariable Cox regression, the hazard ratio was 0.61 (95% CI (0.37, 1.0), p=0.049) with a relative risk reduction of 36.5% with the use of StrataXRT compared with standard moisturizers (Figure 4). No other covariates (systemic therapy, surgery, gender, race, smoking history, BMI, or diabetes history) were significantly associated with grade 2+ dermatitis.

**Figure 5:**
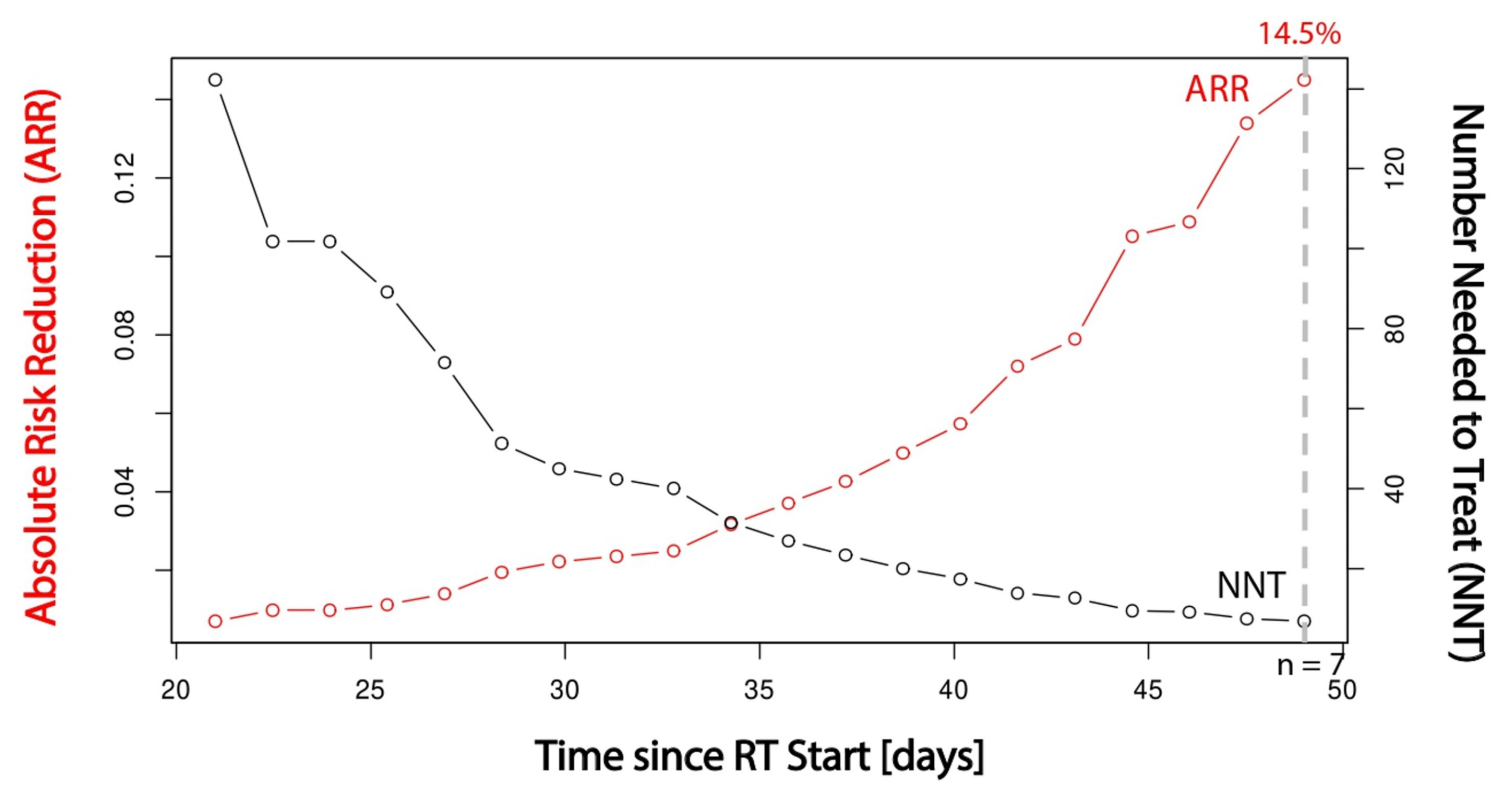
Absolute risk reduction and number needed to treat analyses Absolute risk reduction (ARR) and number needed to treat (NNT) analyses over time showed an ARR of 14.5% at seven weeks after the start of radiation, resulting in an NNT of seven patients treated with StrataXRT to prevent one patient from developing grade 2+ radiation dermatitis.

At 7 weeks after the start of radiation, the use of StrataXRT resulted in an absolute risk reduction of 14.5% in developing grade 2+ radiation dermatitis compared with standard moisturizer alone, corresponding to a number needed to treat of seven patients to spare one grade 2+ radiation dermatitis occurrence (Figure 5). No adverse reactions or events were associated with the use of StrataXRT, as documented.

## Discussion

This retrospective study provides real-world evidence supporting the hypothesis that the use of a novel topical agent, StrataXRT, for patients undergoing definitive radiotherapy for head and neck cancer significantly reduced the incidence of grade 2+ radiation dermatitis compared with standard, over-the-counter, topical moisturizers. These data are derived from practical experience at our institution. There is a 14.5% absolute risk reduction, indicating that for every seven patients treated with StrataXRT, one case of grade 2+ radiation dermatitis can be prevented. There were no adverse reactions or events associated with the use of StrataXRT in our study.

Our approach used a clinical informatics study design to retrospectively evaluate our institutional experience. In 2020, we adopted the use of StrataXRT for nearly all head and neck cancer patients, based on international data from a prospective Australian trial indicating its benefit [5]. After a few years of experience, our informatics approach enabled the rapid identification of patients who received StrataXRT and matching controls. Minimal manual chart review was required, which is a critical advancement in virtual retrospective study design. By having templated notes and using smart string extraction, we were able to automate the aggregation of the data required. Many institutions now offer sandboxed versions of EMR data, and this study highlights the value and utility of analyzing these data using an informatics approach.

These results have an important oncological impact on head and neck cancer patients undergoing radiotherapy. With StrataXRT, patients have an overall delayed onset or progression to grade 2+ radiation dermatitis. Patients treated with StrataXRT have a higher likelihood of making it through the course of radiation without experiencing significant radiation dermatitis. Aside from negatively impacting patients’ quality of life, skin toxicity during radiation is a frequent cause of treatment interruptions and results in an extension of the overall duration of treatment time, which has clinical consequences in tumor control for head and neck cancers. By enabling faster and more comfortable treatment, radiation-specific topical therapies may actually help improve oncological outcomes, as demonstrated previously in studies in which radiation treatment interruptions led to poorer outcomes [15-17].

Although our results are similar to Chan et al.'s 2019 prospective trial in head and neck cancer patients using StrataXRT [5], there are some notable differences. At our institution, we seldomly see grade 3 or higher radiation dermatitis. This may be due to rapid escalation of care upon focal grade 2 moist desquamation. At the detection of patchy moist desquamation, we switch patients to silver sulfadiazine, provide Mepilex paddings to cover the area of exposed skin, and refer them for wound care management. Further, all of our treated patients received intensity-modulated radiation therapy, which also decreases the dose to the skin. Chan et al. reported much higher rates of grade 3 radiation dermatitis, with StrataXRT lowering these rates. Our study shows that StrataXRT was helpful even in delaying grade 2 radiation dermatitis, an important extension of its clinical potential. 

We were able to overcome some challenges in our retrospective design that lacked randomization. Notably, a propensity score matching approach was used to ensure that StrataXRT patients were matched with controls who received similar dosimetry to the skin. This reduced the impact of dosimetric variability, which could have confounded the validity of the effect of StrataXRT. To our knowledge, this was the first radiation dermatitis study that employed propensity score matching on skin dose. 

Nonetheless, there were some key limitations of this study. First, the study was a retrospective analysis of patients who had previously completed treatment. While propensity score matching and covariates in the Cox proportional hazard model were used to best match patients and adjust for variables, this was not a randomized, prospective study. Patients were encouraged to use topical agents twice daily and were reminded during weekly OTVs, though adherence could not be fully ascertained, and the treatments were not applied directly to patients. There was variability in the daily application of the topical treatment across patients in both the control and experimental groups. In spite of these factors, we still observed a treatment effect with the use of StrataXRT. This highlights the real-world experience of using topical agents. Patients anecdotally also often described the texture of StrataXRT cream as smoother to apply, which might have led to better adherence, though this was not directly measured. However, potential better adherence alone may encourage future designs of skin care products for radiotherapy. Further, the measured outcomes were physician-reported CTCAE toxicity scores, not patient-reported outcomes. Data were pulled directly from clinical notes. Even though notes were structured with pre-templated buttons for input and allowed for automatic extraction from the notes, this analysis depends on the quality and accuracy with which physicians appropriately documented toxicity in their clinical notes. Additionally, while StrataXRT delayed the onset of grade 2+ dermatitis, both treatment groups had similar levels of toxicity near the end of treatment, with both requiring equal amounts of escalation of care. Despite this, the delayed onset of grade 2+ radiation dermatitis with StrataXRT improved patients’ comfort and ability to complete radiation without delay. Notably, StrataXRT does require a prescription in the United States, in contrast to over-the-counter topical moisturizers such as Aquaphor®, which may pose socioeconomic and institutional barriers hindering the widespread adoption of StrataXRT. This study and others previously referenced support consideration of health insurance coverage for StrataXRT, as reductions in patient toxicity not only improve health-related quality of life but also potentially reduce the total patients’ medical burden.

Overall, these data reveal that the onset of grade 2+ radiation dermatitis in head and neck cancer patients undergoing radiotherapy can be delayed with a novel, flexible wound dressing, StrataXRT. This investigation encourages further emerging prospective studies, potentially with objective markers of dermatitis that can be captured with short-wave infrared hyperspectral imaging [18]. In addition, combining silicone gel-based topical agents with bacterial decolonization [19] could lead to a robust prevention of radiation dermatitis in patients undergoing head and neck radiotherapy.

## Conclusions

This matched case-control retrospective study demonstrates that StrataXRT, a topical silicone-based film-forming gel, significantly decreased the onset of grade 2+ radiation dermatitis in head and neck cancer patients with an absolute risk reduction of 14.5% (relative risk reduction of 36.5%) compared with standard, over-the-counter topical moisturizers. Our analysis is consistent with prior prospective trials and uses real-world patient data to support the adoption of a novel topical agent providing barrier protection to reduce radiation dermatitis in head and neck cancer patients. Furthermore, we demonstrated the feasibility of running a retrospective study using a clinical informatics approach with automated data extraction from EMRs. This study motivates future clinical informatics investigations of curated EMR data to help assess the efficacy of clinical approaches and regimens after their adoption in routine practice.
